# FRAILTY-FOCUSED ENHANCEMENTS TO SENIORS’ HOSPITAL CARE (FRESH): DOES SPECIALIZED EDUCATION WORK?

**DOI:** 10.1093/geroni/igad104.3362

**Published:** 2023-12-21

**Authors:** Jennifer Peterson, Samantha Fowler, Karla Faig, Kelly Flanagan, Olivia Clancy, Caitlin Robertson, Linda Yetman, Patrick Feltmate

**Affiliations:** Horizon Health Network, Saint John, New Brunswick, Canada; Horizon Health Network, Saint John, New Brunswick, Canada; Horizon Health Network, Saint John, New Brunswick, Canada; Horizon Health, Saint John, New Brunswick, Canada; Horizon Health, Saint John, New Brunswick, Canada; Horizon Health Network, Moncton, New Brunswick, Canada; Horizon Health, Saint John, New Brunswick, Canada; Horizon Health, Fredericton, New Brunswick, Canada

## Abstract

Research suggests that specialized education for nurses decreases frailty and improves functionality in hospitalized older adults. This study explored the impact of a specialized geriatric education program on care delivery for older adults in acute care in 5 hospitals across the province of New Brunswick. A mixed-methods approach with pre- and post- questionnaires was used to explore facilitators and challenges of caring for hospitalized older adults, the knowledge base and experiences of staff, and the impact of providing specialized education. Acute care staff (N=64) participated in a geriatric education intervention and completed pre and post questionnaires. A sub-set (n=26) participated in semi-structured interviews guided by Kirkpatrick’s theoretical framework. Data was collected on mobility, medications, and delirium from patients’ charts (N=99) to identify changes in outcomes pre- and post intervention. Findings revealed that staff had positive attitudes toward caring for older adults; however, their understanding and application of geriatric principles were limited and remained unchanged post-education. There was no significant change in the patient-level measures after the intervention. Interview participants shared that their work environment affects their mindset and workflow, limiting their capacity to deliver the type of care presented in the education sessions. Staff identified the need for specialized education; however, there was no impact on care after participation. Environmental constraints hindered staff from implementing best practices, leading to both practical and psychological challenges to care delivery. These results will inform changes to specialized education programming with the aim of improving care for older adults in hospital.

